# Heritability, SNP- and Gene-Based Analyses of Cannabis Use Initiation and Age at Onset

**DOI:** 10.1007/s10519-015-9723-9

**Published:** 2015-05-19

**Authors:** Camelia C. Minică, Conor V. Dolan, Jouke-Jan Hottenga, René Pool, The Genome of the Netherlands Consortium, Iryna O. Fedko, Hamdi Mbarek, Charlotte Huppertz, Meike Bartels, Dorret I. Boomsma, Jacqueline M. Vink

**Affiliations:** Department of Biological Psychology, Vrije Universiteit Amsterdam, Van der Boechorststraat 1, 1081 BT Amsterdam, The Netherlands

**Keywords:** Cannabis, Initiation, Age at onset, Heritability

## Abstract

Prior searches for genetic variants (GVs) implicated in initiation of cannabis use have been limited to common single nucleotide polymorphisms (SNPs) typed in HapMap samples. Denser SNPs are now available with the completion of the 1000 Genomes and the Genome of the Netherlands projects. More densely distributed SNPs are expected to track the causal variants better. Therefore we extend the search for variants implicated in early stages of cannabis use to previously untagged common and low-frequency variants. We run heritability, SNP and gene-based analyses of initiation and age at onset. This is the first genome-wide study of age at onset to date. Using GCTA and a sample of distantly related individuals from the Netherlands Twin Register, we estimated that the currently measured (and tagged) SNPs collectively explain 25 % of the variance in initiation (SE = 0.088; P = 0.0016). Chromosomes 4 and 18, previously linked with cannabis use and other addiction phenotypes, account for the largest amount of variance in initiation (6.8 %, SE = 0.025, P = 0.002 and 3.6 %, SE = 0.01, P = 0.012, respectively). No individual SNP- or gene-based test reached genomewide significance in the initiation or age at onset analyses. Our study detected association signal in the currently measured SNPs. A comparison with prior SNP-heritability estimates suggests that at least part of the signal is likely coming from previously untyped common and low frequency variants. Our results do not rule out the contribution of rare variants of larger effect—a plausible source of the difference between the twin-based heritability estimate and that from GCTA. The causal variants are likely of very small effect (i.e., <1 % explained variance) and are uniformly distributed over the genome in proportion to chromosomes’ length. Similar to other complex traits and diseases, detecting such small effects is to be expected in sufficiently large samples.

**Electronic supplementary material:**

The online version of this article (doi:10.1007/s10519-015-9723-9) contains supplementary material, which is available to authorized users.

## Introduction

Cannabis is among the drugs with the highest frequency of (ab)use. About 1 in 5 Europeans aged 15–64 reported to have experimented with cannabis. In the United States the prevalence in ages 16–34 was estimated at 51.6 % (European Monitoring Centre for Drugs and Drug Addiction, 2012). Regular cannabis use has been associated with health problems, including mood and anxiety disorders (e.g., Cheung et al. 2010) and chronic bronchitis (Hall 2015; Joshi et al. 2014). Early onset and regular use during adolescence has possible effects on cognitive functioning (e.g., Crean et al. 2011) and predicts diminished educational (Horwood et al. 2010; Lynskey and Hall 2000) and professional attainment (Fergusson and Boden 2008; Volkow et al. 2014). Furthermore, recent evidence suggests that high-potency cannabis use elevates the risk of developing psychotic disorders (Di Forti et al. 2015, 2014). Namely, the odds of showing psychotic symptoms in individuals who declared to have ever used high-potency cannabis are about three times larger than in individuals who declared to have never used cannabis during their lifetime. The risk of showing psychotic symptoms is further elevated if high-potency cannabis is used daily (i.e., OR = 5.4; P = 0.002; Di Forti et al. 2015). About 9 % of those who initiate cannabis use progress to regular use and abuse (e.g., Volkow et al. 2014; Budney et al. 2007). Given the possible adverse effects on health and lifetime outcomes and given its possible role in triggering first-episode of psychosis, it is important to understand the causes of individual differences in the liability to initiate cannabis use.

Twin and family studies have shown that both genetic and environmental factors (both shared by, and specific to, family members) have an important role in the initiation of cannabis use (Kendler and Prescott 1998; van den Bree et al. 1998; Vink et al. 2010). A meta-analysis of twin studies (Verweij et al. 2010) showed that additive genetic factors explain nearly half the variance in liability to initiate cannabis use (i.e., 48 and 40 % of the variance, in females and males, respectively), while the remaining variance is accounted for—almost equally—by shared and unshared environmental factors (both about 30 %).

Among the several attempts to identify genes that explain the heritability of initiation, a linkage study (Agrawal et al. 2008a) failed to identify statistically significant associated genomic regions, although it did identify several suggestive regions on chromosomes 18 and 1. Likewise, a meta-analysis by Verweij et al. (Verweij et al. 2013) combining the results of two genomewide association studies (GWAS) comprising about 10 000 individuals failed to detect common single nucleotide polymorphisms (SNPs) associated with initiation. It should be noted, however, that the association analysis by Verweij and colleagues was limited to common (i.e., minor allele frequency (MAF) > 5 %) HapMap SNPs (Consortium 2010). With the recent completion of large sequencing projects such as the 1000 Genomes (1000G) (Consortium 2012) and the Genome of the Netherlands (Boomsma et al. 2014; The Genome of the Netherlands 2014), more detailed genotypic information has become available in large GWAS samples. Given the availability of denser SNPs, which are expected to be in high linkage disequilibrium (LD) with the causal variants, we aim to extend the search for genetic variants (GVs) implicated in initiation to previously untagged common GVs, and to other (than common) GVs, such as low-frequency variants (1 % < MAF < 5 %). Such low frequency variants have not typically passed the quality control checks. However, the quality of imputation has been improved by recent advances in imputation techniques (Howie et al. 2012). This opens the door to including such GVs into a genome-wide association study.

Furthermore, to date, the approach for finding genes underlying the heritability of cannabis initiation was to focus on the ‘ever/never used’ dichotomy at the expense of the age at which one initiates (i.e., age at onset). Yet, age at onset is a complex trait (Visscher et al. 2001), subject to the influences of both environmental and genetic factors (Lynskey et al. 2003), and may serve as an important proxy for heavy use. Initiation of cannabis use before age 18 is predictive of both experimentation with other drugs (Agrawal et al. 2006; Lynskey et al. 2006), and of escalated drug use (e.g., Lynskey et al. 2003). Among those initiating in adolescence the risk of progression to symptoms of abuse and dependence is higher relative to the general population (i.e., 17 vs. 9 %, respectively; Volkow et al. 2014). Given its relevance as a predictor for escalated use, our second aim is to perform a genomewide search for GVs that give rise to individual differences in age at onset. To model age at onset as a function of genotype we will apply statistical methods based on survival analysis. This approach utilizes all available information on the age at onset among initiateds and takes into account the censored nature of the observations collected in those who did not initiate at the time they were last seen (i.e., they might initiate at a later time point). The approach is expected to show superior power relative to an analysis of the “ever-never” dichotomy or an analysis restricted to those who initiated (see e.g. Kiefer et al. 2013). To our knowledge, a genomewide survival analysis of age at onset of cannabis use has not yet been reported.

The outline of the paper is as follows. First, we estimate the amount of variance in initiation of cannabis use explained collectively by the currently measured SNPs. The purpose of such analysis is to obtain an indication of the total signal in the measured (and tagged) SNPs without identifying individual SNPs. Second, we conduct SNP-based association analyses of initiation and age at onset. Our primary focus is on identifying genes tagged by the SNPs, relevant to our traits. Therefore, next, we incorporate these SNP-based results in two gene-based analyses. These analyses are exploratory, i.e., conducted genomewide.

All analyses are performed in a sample of Dutch families from the Netherlands Twin Register (NTR). To maximize statistical power, imputation of genotypes in the NTR sample was based on two alternative reference panels: the 1000G Phase 1 project reference panel (Consortium 2012) and the reference panel generated by the Genome of the Netherlands (GoNL) project (Boomsma et al. 2014; The Genome of the Netherlands 2014). The GoNL reference panel was derived by sequencing the whole genome of 250 trio-Dutch families and matches therefore the ancestral background of our sample. The GoNL panel is expected to facilitate imputation of variants which are specific to the Dutch population (Boomsma et al. 2014). Furthermore, the use of the GoNL panel is expected to result in higher imputation accuracy relative to the 1000G panel, especially for low frequency GVs (MAF < 5 %) (The Genome of the Netherlands 2014). Such increased accuracy is expected to increase the statistical power to capture the signal in the measured GVs.

## Materials and methods

### Phenotypes

The phenotypic data were obtained in the longitudinal surveys on lifestyle, health, and personality of the NTR (e.g., Boomsma et al. 2002, 2006). The study protocols were approved by the Central Ethics Committee on Research Involving Human Subjects of the VU University Medical Center, Amsterdam. All participants provided informed consent. The study in young twins was approved also by the Central Committee on Research Involving Human Subjects. More details regarding the phenotyping in the NTR study can be found elsewhere (van Beijsterveldt et al. 2013; Willemsen et al. 2013).

#### Initiation of Cannabis use (‘ever/never’)

Initiation was assessed by a multiple choice question (i.e., “At which age did you experiment with cannabis for the first time?”) in the NTR surveys 1993, 1995, 2000, and by an open-ended question (“Have you ever tried hashish or cannabis? If yes, at which age?”) in survey 2009. These surveys were sent to all adult twin families and were returned by 23 597 individuals. In addition, data collection in adolescent twins and sibs which took place since 1987 in age-specific surveys (around age 14 and age 16), included a multiple choice question (“Have you ever used soft drugs such as hashish or cannabis?”) assessing frequency of use (on an eight-category scale ranging from ‘never’ to ‘more than 40 times’) in the whole life, in the last 12 months and in the last 4 weeks. This question was completed by 16 556 participants. The phenotypic data obtained from subjects who reported at more than one time point were checked for consistency, and unreliable measures were discarded. Due to inconsistencies, 284 self-reported measures were dropped. Next, the measurements were collapsed into a dichotomous phenotype (i.e., ever/never used cannabis). Furthermore, we included in the analysis only family members for whom both phenotypes and genotypes were available, i.e., N = 6744 participants. Of these, 5387 individuals reported never to have used cannabis, whereas the remaining 1357 individuals had initiated cannabis use. The age at the time of the last survey ranged from 10.5 to 94 years (mean age = 39.09, SD = 17.45). The participants were clustered within 3479 families varying in size from 1 to 9 family members (i.e., parents, siblings, spouses). More than half of the sample (60.9 %) consisted of females.

#### Age at onset

A subset of the genotyped NTR sample (N = 5148) had declared never to have used cannabis, or declared an age at onset older than 10 years of age in survey 2009 (which included an open ended question on age at onset, see above). Among them, 852 (16.6 %) had initiated cannabis use, whereas 4296 observations had not initiated at the time of data collection (i.e., censored observations). The participants were clustered within 2992 families of sizes varying from 1 to 8 members. Females represented 62.3 % of the sample and the age ranged between 16 and 99 years (mean age = 46.93, SD = 17.54).

### Genotypes

Genotyping was performed based on buccal or blood DNA samples collected in different research projects (see e.g., Willemsen et al. 2010). Imputation was performed based on the 1000G GIANT phase1 panel as a first reference set, and on the GONL version 4 as a second reference set (see Supplementary Methods for details). As best guess genotypes (computed using Beagle, Browning and Yu 2009) were used in the analyses, we applied stringent post imputation quality thresholds on the imputation quality measure (i.e., we retained only SNPs with an imputation quality score above 0.8) and for the Hardy–Weinberg equilibrium test (α = 1 × 10^−4^). Both the imputation quality and Hardy–Weinberg equilibrium (i.e., based on the summed genotype probability counts) were assessed in the phenotyped sample using SNPTEST (Marchini, 2007). The GoNL- and the 1000G-based imputed datasets contained ~6 million well imputed SNPs (i.e., with a mean imputation quality score above 0.96 in both datasets). The association and survival analyses were carried-out by varying the reference panel used for imputation, while including the same phenotyped sample (i.e., 6744 and 5148 participants, respectively). The analyses included no monozygotic twin pairs, because genotypic data were available for only 1 twin of a pair in the GoNL dataset.

### Statistical analyses

#### Estimating the heritability of initiation

We used the Genome-wide Complex Trait Analysis (GCTA) software (Yang et al. 2011) to estimate the amount of variance in initiation explained collectively by the SNPs. The aim of this analysis is to obtain an indication of the total signal in the SNPs, without identifying individual SNPs. Genetic similarity among the phenotyped individuals was computed based on best guess genotypes at 5 928 887 loci observed or imputed using the GoNL reference panel. The analyzed SNPs had a MAF larger than 1 %, imputation quality greater than 0.8 and showed no significant deviation from Hardy–Weinberg equilibrium given α = 1 × 10^−4^. The sample with observed initiation status (N = 6744 related individuals of Dutch ancestry) and the relevant covariates included in the genomewide SNP-based analysis (see below) were also used in the GCTA analysis. Furthermore, one of a pair of closely genetically related individuals (i.e., with an estimated genetic relatedness larger than 0.025) was dropped, which left for the analysis 3616 distantly related individuals. We specified the prevalence as equal to 22 %, value chosen in line with the prevalence of cannabis use estimated in Europeans (European Monitoring Centre for Drugs and Drug Addiction, 2012). Heritability of age at onset was not estimated as GCTA cannot handle survival data. We also investigated the relationship between chromosome length and the amount of variance explained in the trait. Consistent with the model of a polygenic trait, we expect—on average—the longer chromosomes to explain a larger amount of the variance. We tested this in a linear regression (one-tailed test) where we regressed the estimated proportion of variance explained by each chromosome on the chromosome length.

#### Power analysis

We performed a Monte Carlo power analysis to obtain an indication on the size of the genetic effects detectable in our sample. To this end, we simulated 10 000 samples consisting of 3690 families of various configurations reflecting the unbalanced structure of families included in the analyses, i.e., families consisting of singletons, two parents or families comprising sibships sizes 1–6 with 0, 1 or 2 parents. Genotypes in Hardy–Weinberg equilibrium were generated at a locus with a MAF of 0.5 and explaining 1.5 and 1 % variance in the phenotype. The normally distributed phenotype was simulated conditional on the locus and then dichotomized using a cut-off point corresponding to a z-score of 0.85 to mimic the 20 % prevalence of initiation observed in the NTR sample. The correlations between spouses, full siblings and parent-offspring estimated in our sample equaled 0.39, 0.35 and 0.15, respectively. An α = 1 × 10^−8^ was used to assess the power to detect association. To model association we used a generalized equations estimation (GEE) procedure with an exchangeable working correlation matrix and a sandwich correction to correct the standard errors for misspecification of the background model (Minica et al. 2014).

Empirical power analysis showed that our sample affords 45.3 and 87.4 % power to detect GVs explaining 1 and 1.5 % phenotypic variance, respectively (genomewide alpha = 1 × 10^−8^). Relative to the logistic model, the survival model is expected to show superior power especially for locating low frequency causal GVs (see e.g., van der Net et al. 2008). However, the above power computations are informative also for the age at onset phenotype given the large overlap among the samples included in the two analyses and the slightly lower size of the sample we used in the survival analysis.

#### SNP-based association analysis of initiation

To test association, initiation was regressed on the best guess genotype and covariates. The covariates were sex, age at the last survey, the birth cohort (i.e., two birth cohorts containing individuals born between 1951 and 1970 and 1971–1999, respectively, and the 1915–1950 birth cohort as the reference category), 3 principal components to correct for Dutch population substructure (Abdellaoui et al. 2013), and sample specific covariates to account for batch and for chip effects. A GEE (Carey et al. 2012) logistic model was employed. To model the familial relatedness, we used an exchangeable working correlation matrix. This accounts for the familial correlations by means of a single correlation among the family members. The effect of possible misspecification of the familial covariances on the standard errors was corrected by means of a sandwich correction (Minica et al. 2014; Dobson 2002). The sandwich-corrected GEE approach was implemented by using the R-package gee (Carey et al. 2012), accessed from Plink (Purcell et al. 2007) which communicates with R (Team 2013) via the Rserve package (Urbanek 2013).

#### SNP-based survival analysis of age at onset

A Cox proportional hazards regression model was employed to model age at onset as a function of genotype and—as above—of other relevant covariates (i.e., birth cohort, sex, three PCs and study specific covariates). We included this approach as it utilizes all available information on the age of initiation among those who have initiated. It is expected to show superior power relative to an analysis of the “ever-never” dichotomy or an analysis restricted to those who initiated (see e.g. Kiefer et al. 2013). The Cox proportional hazard regression analysis was performed genomewide by accessing the survival R-package (Therneau 2014) from Plink. In fitting the model, we used the cluster option to get sandwich corrected standard errors that are robust to possible misspecification of the familial covariance matrix.

#### Gene-based analyses of initiation and age at onset

Gene-based tests of association with initiation and age at onset were carried out by using the gene-based association test that employs the extended Simes procedure (GATES) implemented in the Knowledge Based Mining System for Genome-wide Genetic Studies software (Li et al. 2011). Specifically, the Simes test extension was employed to combine the P-values of SNPs belonging to the same gene. SNPs were assigned to genes (or to genes’ vicinity, i.e., within a region extended 5 kb at both the 5′ and at the 3′ ends) according to the Human Genome version 19 references. The LD structure was derived based on the GoNL haplotypes and incorporated into the gene-based test as to account for the correlatedness among SNPs within a gene. Lacking prior significant genetic association information related to the cannabis use phenotypes, SNPs were assigned equal weights in the estimation process and the gene-based tests were conducted genomewide for both phenotypes. There were 22 764 genes tested for association with our phenotypes, hence for the gene-based tests the chosen alpha level equaled 0.01/22 746 (i.e., ~4.3 × 10^−7^).

## Results

### Estimating heritability based on genetic relatedness

Results indicate that 25 % [standard error (SE) = 0.088] of the variance on the observed scale in initiation is explained by the SNPs. This amount of variance explained collectively by the SNPs is significantly greater than zero [i.e., likelihood ratio test (LRT) (degrees of freedom = 1) = 8.60, P = 0.0016]. The chromosome-by-chromosome heritability analysis indicated that the largest amount of variance in the trait is explained by chromosome 4 (i.e., the estimate on the observed scale equaled 6.8 %, SE = 0.025, LRT(1) = 7.93, P = 0.002). Chromosome 18 accounted for about 3.6 % (SE = 0.01) of the variance on the observed scale in initiation (LRT(1) = 4.99, P = 0.012).

**Fig. 1:**
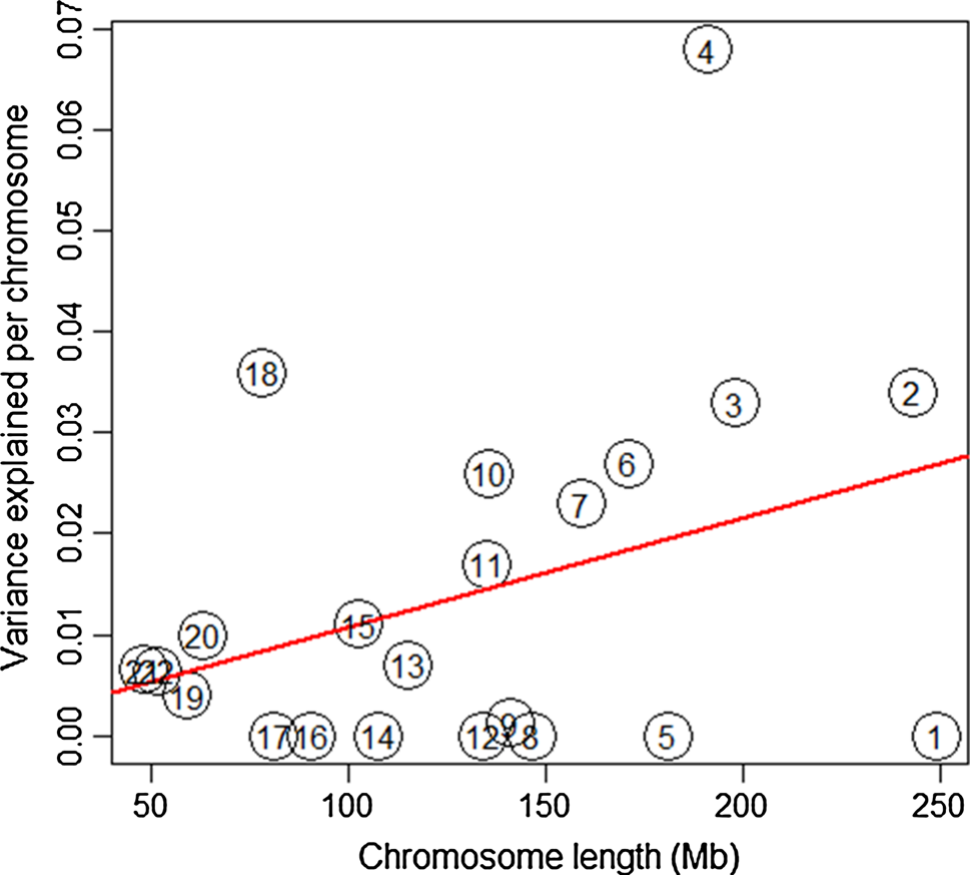
Percent of variance in initiation of cannabis use explained per chromosome relative to chromosome length. The chromosome number is shown in* circles*

We also investigated the relationship between chromosome length and the amount of variance explained (see Supplemental Table S1 for details). We found that chromosome length is significantly associated with proportion of variance explained (one-tailed *t* test(20) = 1.731, P < 0.05). On average longer chromosomes explain a larger percent of variance (Fig. 1).

As shown in Fig. 1, the linear trend is present, notwithstanding the low power to detect variance components attributable to individual chromosomes. The figure demonstrates a trend that is likely to be stronger with increasing sample size. Some parameter estimates hit the lower bound of zero, but this is due to sampling fluctuation (as we illustrate in a small simulation study described in the Supplementary notes). Similar results were reported for other complex traits like intelligence (see e.g., Davies et al. 2011).

### SNP- and gene-based analyses of initiation

SNP-based P-values were obtained in two association analyses of initiation conducted in a sample comprising 6744 participants. Two alternative reference panels—the 1000G and the GoNL, respectively—were used to impute genotypes in our sample. Owing to a better imputation quality (The Genome of the Netherlands 2014), the association signals in the GoNL imputed genotype data were slightly stronger than those obtained based on the 1000G imputed SNPs.^1^ Consequently we took forward these results for the gene-based tests. The P-values for the 5 896 100 GoNL SNPs showed no inflation i.e., the lambda inflation factor equaled 1.019, where a value of 1 indicates no deviation from the expectation of the observed test statistic due to effects of population stratification. The quantile–quantile plot is given in Supplemental Figure S2. The most strongly associated SNP was the low frequency GoNL SNP rs35917943 (MAF < 5 %; P = 1.6 × 10^−7^). The region harboring this SNP is displayed in Supplemental Figure S3 (Pruim et al. 2010). Supplemental Table S2 contains the top SNPs associated with initiation at P < 1 × 10^−5^. Table 1 contains the five genes showing the strongest association signal with initiation along with their functions (according to gene ontology (GO) annotations Ashburner et al. 2000).

**Table 1:** Top five genes showing the strongest association with initiation of cannabis use

Gene name (Gene ID)	Chr	Start position	Number of SNPs assigned to gene	Key SNPs position (rs number)	Gene feature	Key SNPs P-value	Gene P-value	Molecular function according to gene ontology annotation
Zinc finger protein 181 (ZNF181)	19	35225479	2	35221228 (rs35487050)	Upstream	1.6 × 10^−7^	3.7 × 10^−6^	Nucleic acid binding; metal ion binding
microRNA 643 (MIR643)	19	52785049	10	52787471 (rs2434422)	Intronic	3.7 × 10^−6^	3 × 10^−5^	–
				52788044 (rs321908)	Intronic	8.5 × 10^−6^	–	
Zinc finger protein 766 (ZNF766)	19	52772823	41	52787471 (rs2434422)	Intronic	3.7 × 10^−6^	1.1 × 10^−4^	Nucleic acid binding; metal ion binding
				52788044 (rs321908)	Intronic	8.5 × 10^−6^	–	
				52770905 (rs57523152)	Upstream	3.3 × 10^−5^	–	
				52790542 (rs139570481)	Intronic	2.3 × 10^−4^	–	
				52792311 (rs147711278)	Intronic	3.4 × 10^−4^	–	
				52775301 (rs2089275)	Intronic	1 × 10^−2^	–	
Phosphatidylinositol-specific phospholipase C, X domain containing 2 (PLCXD2)	3	111393522	60	111416310 (rs1355767)	Intronic	1.1 × 10^−6^	1.1 × 10^−4^	Phosphoric diester hydrolase activity
				111399209 (rs7651713)	Intronic	1.2 × 10^−6^	–	
				111460129 (rs57628489)	Intronic	1.3 × 10^−2^	–	
				111430969 (rs16858448)	Intronic	1.5 × 10^−2^	–	
				111438443 (rs12637233)	Intronic	1.5 × 10^−2^	–	
				111479048 (rs7643067)	Intronic	1.6 × 10^−2^	–	
				111470751 (rs74571144)	Intronic	1.6 × 10^−2^	–	
				111463864 (rs75923425)	Intronic	1.6 × 10^−2^	–	
				111453629 (rs4682300)	Intronic	1.8 × 10^−2^	–	
				111530499 (rs138770435)	Intronic	2.7 × 10^−2^	–	
				111482694 (rs139568104)	Intronic	3 × 10^−2^	–	
				111443003 (rs9854875)	Intronic	3.2 × 10^−2^	–	
				111449944 (rs7624162)	Intronic	3.2 × 10^−2^	–	
				111514564 (rs11715999)	Intronic	4 × 10^−2^	–	
Prefoldin-like chaperone (URI1)	19	30433145	15	30511638 (rs57192507)	Downstream	2.2 × 10^−5^	1.8 × 10^−4^	Unfolded protein binding
				30465196 (rs7249169)	Intronic	2.7 × 10^−5^	–	
				30509036 (rs73924148)	Downstream	2.7 × 10^−5^	–	
				30442432 (rs77858500)	Intronic	3.1 × 10^−5^	–	
				30432202 (rs58563661)	Intronic	1.1 × 10^−4^	–	
				30418009 (rs61340893)	Intronic	2.9 × 10^−2^	–	

None of these genes had an association P-value below our chosen genomewide level of significance of α = 4.3 × 10^−7^. The three genes with the lowest P-values are Zinc Finger Protein 181 (ZNF181, P = 3.7 × 10^−6^), the non-coding RNA–microRNA 643 (MIR643, P = 3 × 10^−5^) and the Zinc Finger Protein 766 gene (ZNF766, 1.1 × 10^−4^), all located on chromosome 19.

### SNP- and gene-based analyses of age at onset

We conducted two genomewide survival analyses of age at onset in a sample comprising 5148 participants. Similar to the previous analysis, the association signals attained with the genotypes imputed based on the GoNL reference panel were used as input for the gene-based analysis, as these signals were stronger relative to those observed in the 1000G imputed sample (see for a comparison the Manhattan plots, Supplemental Figure S4). As we observed a slight inflation, we corrected the SNP-based P-values (genomic control λ = 1.1171) to prevent potential false positives. Supplemental Figure S5 contains the lambda corrected quantile–quantile plots. The SNP with the strongest association signal was the low-frequency rs142324060 (lambda-corrected P = 7.6 × 10^−8^; MAF < 5 %). The region around the top SNP associated with initiation—rs142324060 on chromosome 5 is displayed in Supplemental Figure S6. The Supplemental Table S3 contains the top SNPs associated with age at onset (P < 1 × 10^−5^).

Table 2 includes the top five genes with the lowest P-values obtained in the gene-based analysis along with their functions (according to GO annotations).

**Table 2:** Top five genes showing the strongest association with age at onset of cannabis use

Gene name (Gene ID)	Chr	Start position	Number of SNPs assigned to gene	Key SNPs position (rs number)	Gene feature	Key SNPs P-value (lambda adjusted)	Gene P-value	Molecular function according to gene ontology annotation
Gem (nuclear organelle) associated protein 5 (GEMIN5)	5	154266975	3	154289310 (rs148816132)	Intronic	1.4 × 10^−5^	4.7 × 10^−4^	Protein binding; snRNA binding
				154272889 (rs816735)	Intronic	0.038	–	
Uncharacterized LOC101927911 (LOC101927911)	17	2865540	9	2871545 (rs4790396)	Intronic	1.6 × 10^−4^	4.7 × 10^−4^	–
Metallothionein 4 (MT4)	16	56598960	13	56598707 (rs141262031)	Upstream	1.9 × 10^−5^	5.2 × 10^−4^	Copper ion binding; zinc ion binding.
				56605477 (rs4784686)	Downstream	0.001	–	
				56596812 (rs71387120)	Upstream	0.003	–	
Kinesin family member 4B (KIF4B)	5	154393259	1	154401490 (rs115299630)	Downstream	3.9 × 10^−5^	5.3 × 10^−4^	Nucleotide binding; DNA binding; microtubule motor activity; ATP binding; microtubule binding;
Peptidylprolyl isomerase G (cyclophilin G) (PPIG)	2	170440849	53	170439011 (rs118138006)	Upstream	3.5 × 10^−5^	5.8 × 10^−4^	Peptidyl-prolyl cis–trans isomerase activity; isomerase activity;
				170444201 (rs78740435)	Intronic	5.7 × 10^−5^	–	
				170437115 (rs12618592)	Upstream	1 × 10^−4^	–	
				170497179 (rs3731675)	Downstream	1.4 × 10^−4^	–	
				170480402 (rs12612841)	Intronic	6.5 × 10^−4^	–	
				170471270 (rs115697204)	Intronic	6.5 × 10^−4^	–	
				170466028 (rs75173877)	Intronic	6.7 × 10^−4^	–	
				170461257 (rs7421113)	Intronic	0.001	–	
				170477394 (rs75968631)	Intronic	0.001	–	

In our exploratory gene-based analysis none of the genes reached the genomewide significance threshold of α = 4.3 × 10^−7^. The genes showing the strongest association with our phenotype were Gem (nuclear organelle) associated protein 5 (GEMIN5) on chromosome 5 (P = 4.7 × 10^−4^) and the uncharacterized LOC101927911 on chromosome 17 (P = 4.7 × 10^−4^), followed by the Metallothionein 4 (MT4) on chromosome 16 (P = 5.2 × 10^−4^). The SNP with the strongest association signal—the rs142324060 (lambda-corrected P = 7.6 × 10^−8^) was not assigned to a gene in the GATES analysis.

## Discussion

The aim of the study was to explore the contribution of GVs to initiation of cannabis use and age at onset. Using GCTA and a sample of distantly related individuals from the NTR, we estimated that the genomewide SNPs collectively explain 25 % (SE = 0.088; P = 0.0016) of the variance in initiation. Although lower than the twin-based heritability estimate (i.e., of about 44 % (95 % CI [16 %,74 %], Vink et al. 2010), our estimate provides an indication of the total signal in the currently measured (and tagged) SNPs, confirming that initiation of cannabis use is a heritable trait. The remaining variance (up to 44 %) may, in part, be attributable to rare variants, weakly correlated with the measured SNPs (Visscher et al. 2010). Our estimate is larger than that reported by Verweij and colleagues, namely 6 % (95 % CI [0 %, 26 %], P-value = ns). A possible reason for this difference is that we use more densely distributed SNPs. In addition to the common SNPs overlapping with the HapMap SNPs used by Verweij and colleagues (about 2.4 million common SNPs with MAF > 5 %), we included into analysis previously untagged common GVs, and other (than common) GVs, such as low-frequency variants (about 6 million SNPs having MAF > 1 %). More densely distributed SNPs are expected to be in higher LD with the causal variants, and so, to provide a more accurate heritability estimate (Visscher et al. 2010).

The chromosome-by-chromosome analyses showed that, on average, longer chromosomes account for a larger amount of variance in initiation. This result lends support to the conclusion that initiation is highly polygenic. The largest amount of variance is explained by chromosome 4 (6.8 %; P = 0.002), followed by chromosome 18 (3.6 %; P = 0.012). Regions on both chromosome 4 and 18 have been reported to play a role in cannabis use and other addiction phenotypes. For instance, regions on chromosome 4 harboring the GABRA cluster of genes were identified in a linkage study by Agrawal et al. (Agrawal et al. 2008b) as plausibly associated with a cannabis abuse and dependence phenotype. Another linkage study (Prescott et al. 2006) provided strong evidence for a large region on chromosome 4 to be involved in alcohol dependence (P = 2.1 × 10^−6^), the same region being also reported by Uhl et al. to be associated with illicit drug abuse (Uhl et al. 2002). Regions on chromosome 18 were suggested to harbor GVs potentially associated with initiation of cannabis use (Agrawal et al. 2008a), methamphetamine abuse (Lee et al. 2014) and alcohol dependence (Prescott et al. 2006). However, when tested individually, none of the GVs achieved an association P-value less than the adapted (i.e., for multiple testing) alpha of 1 × 10^−8^.

We further explored how our results compare with previously published ones. Using the SNP effect concordance method (Nyholt 2014) and the NTR as a replication sample, we checked whether there is an excess of SNPs showing concordant effects in the meta-analysis by Verweij et al. (2013) and in our analysis. Of the 2 110 385 HapMap SNPs tested in both samples, we selected for the comparison 25 204 independent HapMap SNPs (r^2^ > 0.1) that showed the most significant association P-values in the meta-analysis sample. Although we compare summary results for the same phenotype (cannabis initiation) such an analysis is similar in scope to a search for significant pleiotropic effects (genetic overlap): we aimed to single out sets of SNPs showing concordant effects in the two samples beyond what is expected by chance. Concordance of effects was assessed by exact binomial tests. We observed no significant excess of SNPs with concordant effects in the two datasets. It is possible that the effects of the causal variants are too small to be accurately captured by the two samples. It is also likely that the causal GVs were imperfectly tagged by the selected SNPs (e.g., because they have a lower MAF than the selected SNPs), and this further decreased the estimation precision in both samples.

None of the tested genes achieved genomewide significance (P < ~4.3 × 10^−7^). However, our results have pinpointed several possible candidate genomic regions, likely to have a bearing on the early stage of cannabis use. To name a few, the ZNF181 and the ZNF766 genes, both located on chromosome 19, yielded the strongest association signal in the gene-based analysis of initiation (i.e., P = 3.7 × 10^−6^, 1.1 × 10^−4^, respectively). According to the GO annotations, ZNF181 and ZNF766 are functional genes belonging to the zinc finger family of genes, being involved in nucleic acid binding and metal ion binding. The most strongly associated genes with age at onset were the protein coding genes GEMIN5 (P = 4.7 × 10^−4^) on chromosome 5 and MT4 on chromosome 16 (P = 5.2 × 10^−4^). GEMIN5 plays a role in protein binding and snRNA binding, whereas MT4 is involved in copper ion and zinc ion binding. The role these genes play in initiation and age at onset has yet to be clarified, as none have been previously reported to be associated with cannabis use or other addiction phenotypes.

To our knowledge this is the first genomewide survival analysis of age at onset of cannabis use to date. The survival modeling approach appears to be appropriate and computationally tractable given the detailed genotypic data currently available (an example dataset and annotated scripts to run such an analysis can be found at http://cameliaminica.nl/research.php). Clearly, further research on the genetic basis of age at onset would be of interest as the trait may serve as a proxy for both heavy use and experimentation with other drugs.

Our study detected association signal in the measured SNPs. A comparison with prior SNP-heritability estimates suggests that at least part of the signal is likely coming from previously untyped common and from low frequency variants. The lack of genomewide significant results for the single variant and gene-based association tests suggests that initiation is a polygenic trait characterized by variants of very small effect (i.e., <1 % explained phenotypic variance). The causal variants are likely distributed over much of the genome, in proportion to the chromosomes’ length. Our results do not rule out the contribution of rare variants of larger effect imperfectly tracked by the measured SNPs—a plausible source of the difference between the twin-based heritability estimate and that from GCTA. Powerful analytic strategies and very large samples combined with considering the contribution of rare variants (MAF < 1 %) will allow one to further understand the causes of individual differences in the liability to initiate cannabis use.

## Electronic supplementary material

Supplementary material 1 (DOCX 1311 kb)

## Footnotes

1 We include for a comparison the Manhattan plots of the association results based on data imputed using the two alternative reference panels (see Supplemental Figure S1). They illustrate the gains in power—in terms of improved association signals - conferred by a population specific reference panel relative to the 1000 Genomes. These results are likely informative for groups contemplating the use of alternative panels to impute GWAS samples.
