# Supplementary material for: Heritability, SNP- and Gene-Based Analyses of Cannabis Use Initiation and Age at Onset
Source: Behav Genet. 2015 May 19;45(5):503–13. doi: 10.1007/s10519-015-9723-9 (PMC4561059; doi:10.1007/s10519-015-9723-9)

**SUPPLEMENTARY MATERIAL**

**Supplementary Methods**

**Genotyping and imputation in the NTR sample**

Genotyping in the NTR sample was performed based on buccal or blood DNA samples collected in different research projects (for details see e.g., ([Willemsen et al., 2010](#_ENREF_45))) using various genotyping platforms. For genotype calling we used platform specific software. We removed from each platform SNPs that failed the subsequent liftover to Human Genome version 19 references (build 37). Namely, we dropped SNPs that were not mapped, or lacked matches, or had ambiguous positions. Following strand alignment with the 1000 Genomes GIANT phase1 release v3 20101123 SNPs INDELS SVS ALL panel as a first reference set, and with the GONL version 4 as a second reference set, data from each platform underwent further quality checks. Specifically, we discarded SNPs not in Hardy-Weinberg equilibrium (α =10^-5^), and SNPs either showing mismatches with one of the reference sets or having a low call rate (less than 95%). Furthermore, we removed SNPs whose allele frequency differed more than 20% relative to each reference set, or had a minor allele frequency below 1%. To prevent incorrect strand alignment, we also removed SNPs with C/G and A/T allele combinations having a minor allele frequency between 0.35 and 0.5. SNPs typed multiple times showing less than 99% concordance rate were also dropped. Next, individuals displaying either high or very low homozygozity rates (i.e., the estimated F inbreeding coefficient was either larger than 0.10 or lower than -0.10, indicating deviation from expectation of the number of observed homozygous genotypes) or individuals having genotype missing rates above 10% were excluded. In addition, we discarded individuals whose estimated identity by state (IBS) sharing mismatched their expected IBS given the NTR pedigree structure. The above quality checks were then performed on the dataset resulted from merging genotype data typed on different platforms. 12.240 unique DNA samples were taken forward for imputation. MACH 1.0 ([Li et al., 2010](#_ENREF_25)) was used for phasing and imputing cross-platform missing SNPs and Minimac ([Li et al., 2010](#_ENREF_25)) was used for imputing genotypes in the phased data. SNPs having minor allele frequency lower than 1% were removed from the imputed dataset.

**Supplementary Notes**

**Simulation study**

We investigated the relationship between chromosome length and the amount of variance explained. As expected for highly polygenic traits, we found that chromosome length is significantly associated with proportion of explained variance, with longer chromosomes explaining on average a larger percent of variance. Some parameter estimates such as e.g., the variance component for chromosome 1, despite its largest size, happened to hit the lower bound of zero. Assuming that the causal variants are uniformly distributed over autosomal chromosomes, we conjectured that the zero variances attributable to some individual chromosomes are due to sampling fluctuation. To demonstrate this, we conducted a small simulation study. Using GCTA we generated 10 phenotypic samples based on the real genotypes observed in the NTR sample and on the parameter values estimated in the real data. Namely, the trait heritability equaled 25% and the SNPs were assigned the effects obtained in the genomewide association study of initiation. Given the simulated phenotypes and the real genotypes, we estimated the variance explained collectively by the SNPs on chromosome 1. As in the real data analysis, we used the --keep option to use in the estimation a list of 3659 distantly related individuals. We set the prevalence to equal 0.22. Table 1 contains the results, with the estimates obtained in the real data included in the first row.

Table 1: Estimates of the variance explained by the SNPs on chromosome 1 in the NTR sample (cases = 656, controls=3003). The trait heritability equaled 25% and the user specified prevalence equalled 0.22. In red bold are given the results for the samples in which the variance component attributable to chromosome 1 hit the lower bound of zero.

| Chromosome  1 | Variance explained  on the observed scale  (SE) | Variance explained  on the liability scale  (SE) | LRT  (df) | P-value |
| --- | --- | --- | --- | --- |
| **REAL DATA** | **0.000001**  **(0.026)** | **0.000002**  **(0.059)** | **LRT (1)=0** | **P=0.5** |
| SIMULATED | 0.010330  (0.026) | 0.022  (0.058451) | LRT (1)=0.15 | P=0.345 |
| SIMULATED | 0.063  (0.028) | 0.140  (0.063) | LRT(1)=5.45 | P=0.009 |
| SIMULATED | 0.041  (0.027) | 0.091  (0.061) | LRT(1)=2.39 | P=0.06 |
| SIMULATED | 0.022  (0.027) | 0.049  (0.060) | LRT(1)=0.702 | P=0.201 |
| SIMULATED | 0.046  (0.027) | 0.102  (0.060) | LRT(1)=3.35 | P=0.033 |
| **SIMULATED** | **0.000001**  **(0.026)** | **0.000002**  **(0.059)** | **LRT(1)=0** | **P=0.5** |
| SIMULATED | 0.0056  (0.026) | 0.0125  (0.059) | LRT(1)=0.043 | P=0.417 |
| SIMULATED | 0.029  (0.026) | 0.065  (0.059) | LRT(1)=1.359 | P=0.121 |
| **SIMULATED** | **0.000001**  **(0.026)** | **0.000002**  **(0.0576)** | **LRT(1)=0** | **P=0.5** |
| SIMULATED | 0.037  (0.028) | 0.083005  (0.061) | LRT(1)=1.891 | P=0.084 |

Note that in 2 out of the 10 simulated samples, the SNPs on the chromosome 1 explain zero variance. In the remaining ones the parameter estimate is different from zero, fluctuating from 0.05% to 6%. This fluctuation in estimates is expected as it largely depends on the size of the sample (which is small in our case). Although small, the standard errors are highly relevant in this context because the genetic relationships estimated based on the SNPs on one chromosome are necessarily very small (as they are calculated in pairs of distantly related individuals; see ([Visscher et al. 2010](#_ENREF_42))).

More importantly, despite the large sampling fluctuation, we nicely captured the linear relationship between the chromosome length and amount of variance explained. This result lends support to the conclusion that cannabis use is a highly polygenic trait.

**Supplementary Tables**

Table S1. Estimates of the variance explained in the initiation of cannabis use by each of the 22 autosomal chromosomes. These estimates were obtained by using the Genome-wide Complex Trait Analysis (GCTA) software (Yang et al. 2010). For each analysis the sample consisted of N=3659 unrelated individuals from the Netherlands Twin Register who had observed initiation of cannabis use status. This list of individuals was provided as input for each analysis by using the --keep option. The specified prevalence of initiation of cannabis use was 22%, whereas the prevalence in the analyzed sample (of unrelated individuals) was 18%.

| Chromosome | Variance explained  on the observed scale  (SE) | Variance explained  on the liability scale  (SE) | LRT  (df) | P-value |
| --- | --- | --- | --- | --- |
| 1 | 0.000001  (0.026) | 0.000002  (0.059) | LRT (1)=0 | P=0.5 |
| 2 | 0.034  (0.027) | 0.078  (0.062) | LRT(1)=1.681 | P=0.09 |
| 3 | 0.033  (0.024) | 0.075  (0.054) | LRT(1)=2.224 | P=0.06 |
| 4 | 0.068  (0.025) | 0.157  (0.059) | LRT(1)=7.933 | P=0.002 |
| 5 | 0.000001  (0.021) | 0.000002  (0.049) | LRT(1)=0 | P=0.5 |
| 6 | 0.027  (0.023) | 0.063  (0.054) | LRT(1)=1.396 | P=0.11 |
| 7 | 0.023  (0.022) | 0.053  (0.051) | LRT(1)=1.113 | P=0.14 |
| 8 | 0.000001  (0.020) | 0.000002  (0.046) | LRT(1)=0 | P=0.5 |
| 9 | 0.0013  (0.020) | 0.003  (0.046) | LRT(1)=0.004 | P=0.47 |
| 10 | 0.026  (0.021) | 0.060  (0.048) | LRT(1)=1.794 | P=0.09 |
| 11 | 0.017  (0.018) | 0.039  (0.041) | LRT(1)=1.153 | P=0.14 |
| 12 | 0.000001  (0.019) | 0.000002  (0.045) | LRT(1)=0 | P=0.5 |
| 13 | 0.007  (0.018) | 0.016  (0.041) | LRT(1)=0.162 | P=0.34 |
| 14 | 0.000001  (0.016) | 0.000002  (0.038) | LRT(1)=0 | P=0.5 |
| 15 | 0.011  (0.016) | 0.025  (0.037) | LRT(1)=0.482 | P=0.24 |
| 16 | 0.000001  (0.018) | 0.000002  (0.041) | LRT(1)=0 | P=0.5 |
| 17 | 0.000001  (0.015) | 0.000002  (0.034) | LRT(1)=0 | P=0.5 |
| 18 | 0.036  (0.018) | 0.082  (0.041) | LRT(1)=4.994 | P=0.012 |
| 19 | 0.004  (0.012) | 0.010  (0.028) | LRT(1)=0.171 | P=0.33 |
| 20 | 0.010  (0.015) | 0.024  (0.035) | LRT(1)=0.594 | P=0.22 |
| 21 | 0.0065  (0.012) | 0.014  (0.028) | LRT(1)=0.284 | P=0.29 |
| 22 | 0.0064  (0.012) | 0.0147  (0.028) | LRT(1)=0.297 | P=0.29 |

Abbreviations: SE, standard error; LRT, likelihood ratio test; df, degrees of freedom;

Table S2. Top GoNL SNPs associated with cannabis use initiation. The analysis was performed by using a gee model with an exchangeable working correlation matrix. Selection of SNPs was made using a cut-off P-value of 10^-5^.

| SNP | Chromosome | Position | Effect  allele | Non-effect  allele | Beta | SE | P-value |
| --- | --- | --- | --- | --- | --- | --- | --- |
| rs35917943 | 19 | 35147183 | C | T | .77 | .15 | 1.62E-007 |
| rs35487050 | 19 | 35221228 | C | A | .81 | .16 | 1.68E-007 |
| rs35760174 | 19 | 35221582 | C | G | .76 | .15 | 7.04E-007 |
| rs1355767 | 3 | 111416310 | A | G | -.25 | .05 | 1.16E-006 |
| rs7651713 | 3 | 111399209 | T | C | -.27 | .05 | 1.29E-006 |
| rs2656620 | 16 | 78913387 | A | C | .23 | .05 | 1.58E-006 |
| rs16948735 | 16 | 78916152 | A | C | .24 | .05 | 1.88E-006 |
| rs6835174 | 4 | 5976104 | T | C | .34 | .07 | 3.28E-006 |
| rs4243162 | 16 | 78918109 | G | A | .23 | .05 | 3.36E-006 |
| rs16837971 | 4 | 5977133 | C | A | .34 | .07 | 3.64E-006 |
| rs2434422 | 19 | 52787471 | C | T | -.47 | .10 | 3.78E-006 |
| rs2656629 | 16 | 78911833 | T | A | .23 | .05 | 4.19E-006 |
| rs2656628 | 16 | 78912070 | A | C | .23 | .05 | 4.57E-006 |
| rs11121321 | 1 | 9154622 | T | C | .82 | .18 | 4.76E-006 |
| rs316577 | 5 | 2294688 | A | G | -.23 | .05 | 4.81E-006 |
| rs8049189 | 16 | 78926895 | C | T | .21 | .05 | 4.93E-006 |
| rs4516655 | 4 | 5975378 | A | G | .34 | .07 | 5.22E-006 |
| rs2656626 | 16 | 78912114 | G | C | .23 | .05 | 5.39E-006 |
| rs2656618 | 16 | 78913607 | T | G | .22 | .05 | 5.39E-006 |
| rs4887990 | 16 | 78920901 | G | A | .22 | .05 | 5.52E-006 |
| rs4481129 | 3 | 111405911 | T | C | -.25 | .06 | 5.70E-006 |
| rs456840 | 5 | 2294552 | C | T | -.23 | .05 | 5.76E-006 |
| rs2656619 | 16 | 78913461 | A | G | .22 | .05 | 5.78E-006 |
| rs9510661 | 13 | 23851799 | C | A | -.39 | .09 | 5.80E-006 |
| rs12239636 | 1 | 9155701 | T | C | .81 | .18 | 5.81E-006 |
| rs9510662 | 13 | 23852058 | T | C | -.40 | .09 | 5.88E-006 |
| rs222548 | 6 | 95211552 | T | C | -.60 | .13 | 6.09E-006 |
| rs17706982 | 16 | 78918983 | G | C | .22 | .05 | 6.38E-006 |
| rs11809230 | 1 | 70084797 | T | C | .29 | .06 | 6.43E-006 |
| rs2656621 | 16 | 78913315 | A | G | .22 | .05 | 6.50E-006 |
| rs35751268 | 6 | 149113146 | T | C | .23 | .05 | 6.53E-006 |
| rs1106616 | 16 | 78910841 | C | T | .22 | .05 | 7.06E-006 |
| rs316578 | 5 | 2294533 | A | G | -.23 | .05 | 7.08E-006 |
| rs4887991 | 16 | 78921063 | G | A | .22 | .05 | 7.39E-006 |
| rs112885004 | 4 | 5983270 | A | T | .32 | .07 | 7.40E-006 |
| rs2656622 | 16 | 78913164 | G | C | .22 | .05 | 7.67E-006 |
| rs7558233 | 2 | 23681924 | T | A | .48 | .11 | 7.95E-006 |
| rs456963 | 5 | 2294550 | G | A | -.22 | .05 | 7.99E-006 |
| rs7020651 | 9 | 22972837 | A | C | .38 | .08 | 8.00E-006 |
| rs2656624 | 16 | 78912730 | A | G | .22 | .05 | 8.35E-006 |
| rs7540133 | 1 | 70069253 | C | T | .27 | .06 | 8.46E-006 |
| rs28581422 | 7 | 121258371 | C | T | -.65 | .15 | 8.51E-006 |
| rs28592962 | 7 | 121258514 | C | A | -.65 | .15 | 8.51E-006 |
| rs57360413 | 7 | 121258513 | G | A | -.65 | .15 | 8.51E-006 |
| rs28480595 | 19 | 52787905 | C | G | -.43 | .10 | 8.57E-006 |
| rs321908 | 19 | 52788044 | C | T | -.43 | .10 | 8.57E-006 |
| rs2656623 | 16 | 78912995 | G | A | .22 | .05 | 9.54E-006 |
| rs9530740 | 13 | 78741106 | G | C | -.21 | .05 | 9.73E-006 |
| rs1079634 | 16 | 78911134 | G | T | .22 | .05 | 9.83E-006 |

Table S3. Top GoNL SNPs associated with age of onset in the Netherlands Twin Register sample. The analysis was performed by using a Cox regression model and a sandwich correction for the standard errors. Selection of SNPs was performed by using a cut-off lambda adjusted P-value of 10^-5^.

| SNP | Chromosome | Position | Effect  allele | Non-effect  allele | Beta | SE | P-value |
| --- | --- | --- | --- | --- | --- | --- | --- |
| rs142324060 | 5 | 95425757 | G | A | .68 | .11 | 7.66E-008 |
| rs78505392 | 5 | 95422966 | C | G | .58 | .10 | 2.16E-007 |
| rs12003072 | 9 | 86771161 | A | C | .52 | .09 | 3.04E-007 |
| rs77097806 | 5 | 95456735 | A | G | .56 | .10 | 3.54E-007 |
| rs6879646 | 5 | 95450187 | A | G | .57 | .10 | 3.61E-007 |
| rs4613744 | 5 | 95451494 | C | T | .55 | .10 | 5.07E-007 |
| rs60218730 | 5 | 95492765 | G | T | .59 | .11 | 5.98E-007 |
| rs74305417 | 9 | 86779774 | C | G | .52 | .09 | 6.20E-007 |
| rs142981069 | 18 | 58826022 | G | A | .47 | .09 | 7.25E-007 |
| rs12386084 | 18 | 58827145 | C | G | .47 | .09 | 7.25E-007 |
| rs117918936 | 18 | 58828323 | G | A | .47 | .09 | 7.25E-007 |
| rs2160801 | 18 | 58829024 | T | A | .47 | .09 | 7.25E-007 |
| rs145424173 | 18 | 58829597 | T | C | .47 | .09 | 7.25E-007 |
| rs117538409 | 18 | 58830942 | G | C | .47 | .09 | 7.25E-007 |
| rs17817245 | 18 | 58832135 | A | G | .47 | .09 | 7.25E-007 |
| rs140206809 | 18 | 58833215 | A | G | .47 | .09 | 7.25E-007 |
| rs117692712 | 18 | 58834506 | T | G | .47 | .09 | 7.25E-007 |
| rs17817423 | 18 | 58835462 | C | T | .47 | .09 | 7.25E-007 |
| rs9916935 | 18 | 58835931 | T | C | .47 | .09 | 7.25E-007 |
| rs192013604 | 18 | 58838324 | T | C | .47 | .09 | 7.25E-007 |
| rs117471640 | 18 | 58838402 | A | G | .47 | .09 | 7.25E-007 |
| rs78456402 | 9 | 86781900 | C | A | .50 | .09 | 9.09E-007 |
| rs11998981 | 9 | 86783107 | T | C | .50 | .09 | 9.09E-007 |
| rs79236058 | 5 | 95478830 | G | A | .57 | .10 | 9.59E-007 |
| rs117659340 | 18 | 58859359 | A | C | .46 | .08 | 1.15E-006 |
| rs2059585 | 18 | 58860892 | T | A | .46 | .08 | 1.15E-006 |
| rs2059586 | 18 | 58860942 | G | C | .46 | .08 | 1.15E-006 |
| rs117111407 | 18 | 58869269 | C | T | .45 | .08 | 1.59E-006 |
| rs77170674 | 18 | 58869411 | G | A | .45 | .09 | 1.90E-006 |
| rs188886252 | 18 | 58869495 | A | G | .45 | .09 | 1.90E-006 |
| rs116866095 | 18 | 58869572 | C | T | .45 | .09 | 1.90E-006 |
| rs140158414 | 18 | 58872063 | G | T | .45 | .09 | 1.90E-006 |
| rs190532486 | 18 | 58873959 | A | T | .45 | .09 | 1.90E-006 |
| rs117815864 | 18 | 58875399 | T | C | .45 | .09 | 1.90E-006 |
| rs145084328 | 18 | 58876782 | T | A | .45 | .09 | 1.90E-006 |
| rs141558278 | 18 | 58877206 | C | A | .45 | .09 | 1.90E-006 |
| rs10520189 | 4 | 171641235 | A | G | .29 | .05 | 1.99E-006 |
| rs76280858 | 5 | 17876401 | T | C | .21 | .04 | 2.11E-006 |
| rs77551987 | 5 | 95493213 | G | A | .58 | .11 | 2.24E-006 |
| rs17240113 | 18 | 58879356 | C | T | .45 | .09 | 2.49E-006 |
| rs78152895 | 5 | 17844797 | C | G | .21 | .04 | 2.90E-006 |
| rs76639472 | 18 | 58841101 | A | G | .44 | .08 | 2.94E-006 |
| rs117798039 | 18 | 58841135 | T | C | .44 | .08 | 2.94E-006 |
| rs140032812 | 18 | 58843167 | A | C | .44 | .08 | 2.94E-006 |
| rs117046191 | 18 | 58846121 | T | C | .44 | .08 | 2.94E-006 |
| rs76021144 | 18 | 58849162 | A | T | .44 | .08 | 2.94E-006 |
| rs149836886 | 18 | 58849335 | T | A | .44 | .08 | 2.94E-006 |
| rs78373721 | 18 | 58849384 | A | T | .44 | .08 | 2.94E-006 |
| rs11877018 | 18 | 58849456 | G | A | .44 | .08 | 2.94E-006 |
| rs9951061 | 18 | 58849730 | A | G | .44 | .08 | 2.94E-006 |
| rs9951700 | 18 | 58849751 | A | C | .44 | .08 | 2.94E-006 |
| rs17817765 | 18 | 58850622 | G | T | .44 | .08 | 2.94E-006 |
| rs9967035 | 18 | 58850924 | G | A | .44 | .08 | 2.94E-006 |
| rs9954454 | 18 | 58850962 | A | G | .44 | .08 | 2.94E-006 |
| rs117929008 | 18 | 58852748 | T | G | .44 | .08 | 2.94E-006 |
| rs12104065 | 18 | 58853017 | T | G | .44 | .08 | 2.94E-006 |
| rs75712581 | 18 | 58853832 | T | C | .44 | .08 | 2.94E-006 |
| rs17067915 | 18 | 58853958 | T | G | .44 | .08 | 2.94E-006 |
| rs28377454 | 18 | 58854375 | T | C | .44 | .08 | 2.94E-006 |
| rs10513923 | 18 | 58856268 | G | A | .44 | .08 | 2.94E-006 |
| rs78818781 | 5 | 17874674 | A | C | .21 | .04 | 2.96E-006 |
| rs76395821 | 5 | 17878062 | T | C | .21 | .04 | 3.73E-006 |
| rs114177134 | 5 | 17849701 | G | A | .21 | .04 | 3.79E-006 |
| rs181704351 | 1 | 70147866 | T | C | .46 | .09 | 4.75E-006 |
| rs17240163 | 18 | 58879630 | G | A | .43 | .08 | 5.06E-006 |
| rs114403726 | 5 | 154056080 | A | G | .44 | .09 | 5.65E-006 |
| rs141854787 | 16 | 49786258 | T | C | .35 | .07 | 6.46E-006 |
| rs10925507 | 1 | 237913281 | A | G | .27 | .05 | 7.58E-006 |
| rs117711289 | 18 | 58846334 | A | G | .42 | .08 | 7.77E-006 |
| rs181934145 | 5 | 95504979 | C | T | .54 | .11 | 8.67E-006 |
| rs186425099 | 5 | 95506661 | T | C | .54 | .11 | 8.67E-006 |
| rs57801175 | 5 | 95507884 | T | G | .54 | .11 | 8.67E-006 |
| rs116578151 | 5 | 95511750 | G | A | .54 | .11 | 8.67E-006 |
| rs78920411 | 5 | 95511801 | C | T | .54 | .11 | 8.67E-006 |
| rs191911126 | 1 | 69990841 | A | G | .45 | .09 | 9.55E-006 |

**Supplementary figures**

Figure S1: Manhattan plots for the initiation of cannabis use analysis. The analysis included same phenotyped sample from the Netherlands Twin Register (N=6744 individuals) with genotypes imputed based on (a) the 1000 Genomes project reference panel and based on (b) the Genome of the Netherlands (GoNL) project reference panel.

a.


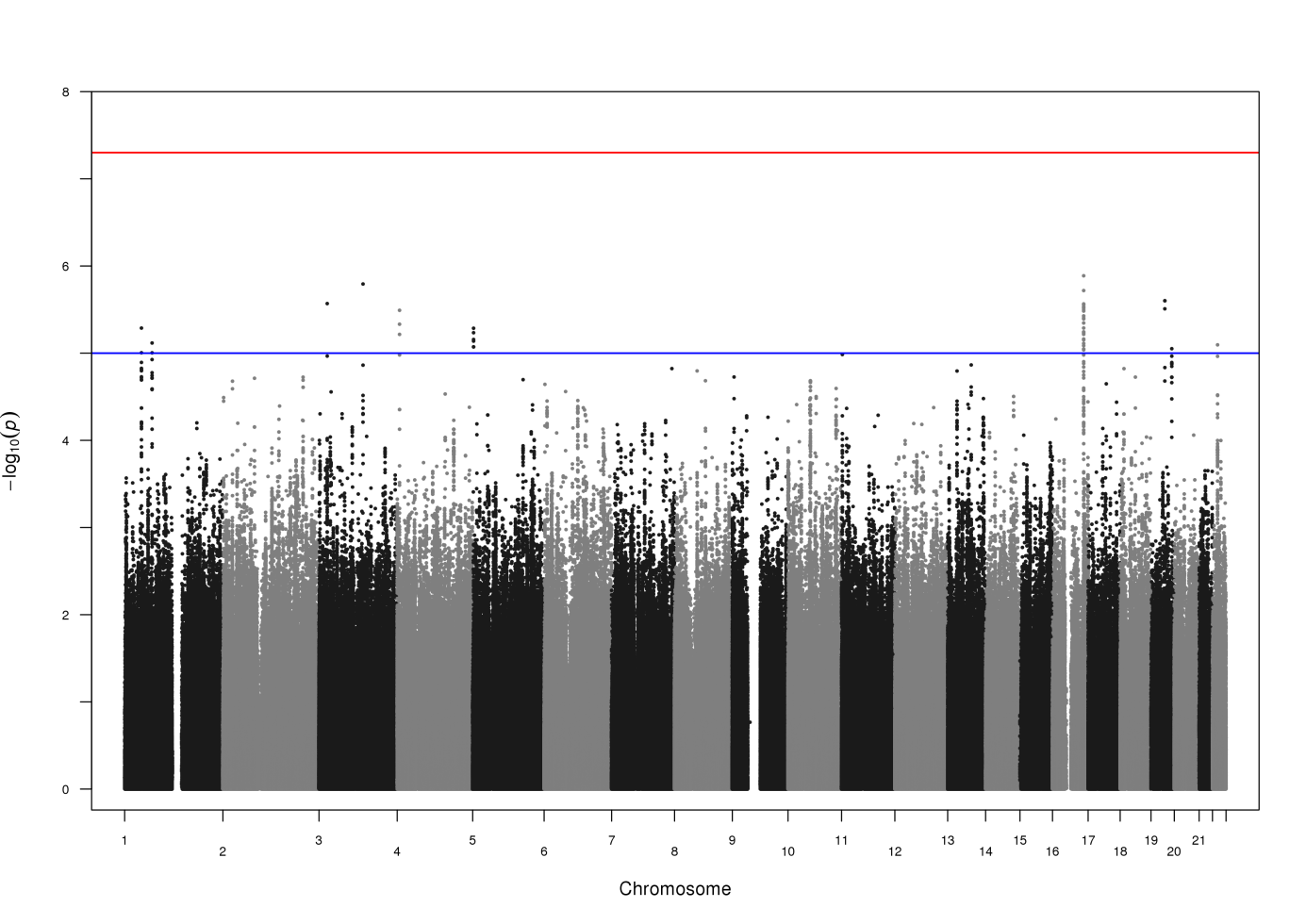


b.

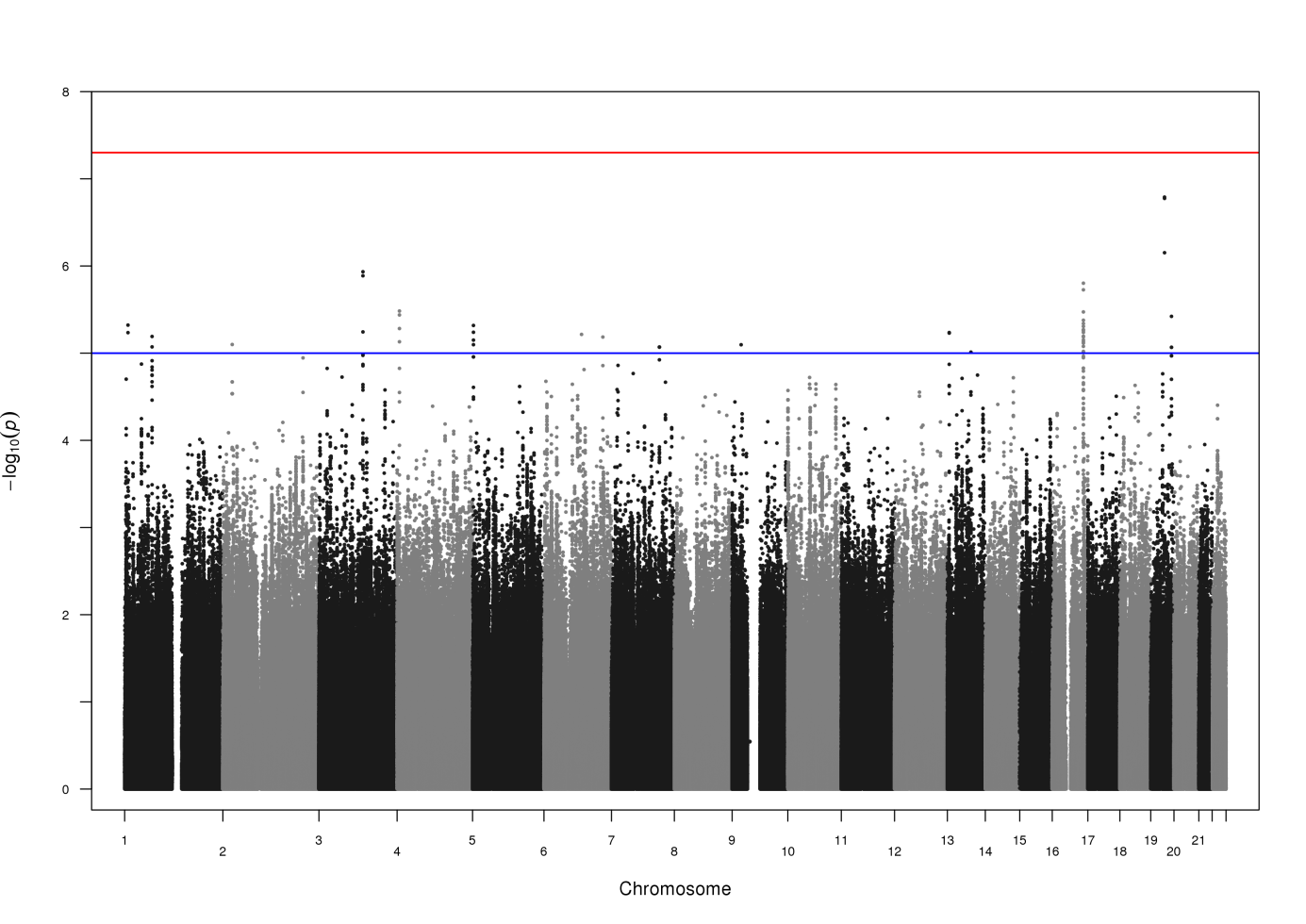


Figure S2: Quantile-quantile plots for the initiation of cannabis use analysis. The analysis included same phenotyped sample from the Netherlands Twin Register (N=6744 individuals) with genotypes imputed based on (a) the 1000 Genomes project reference panel and based on (b) the Genome of the Netherlands (GoNL) project reference panel.

a.


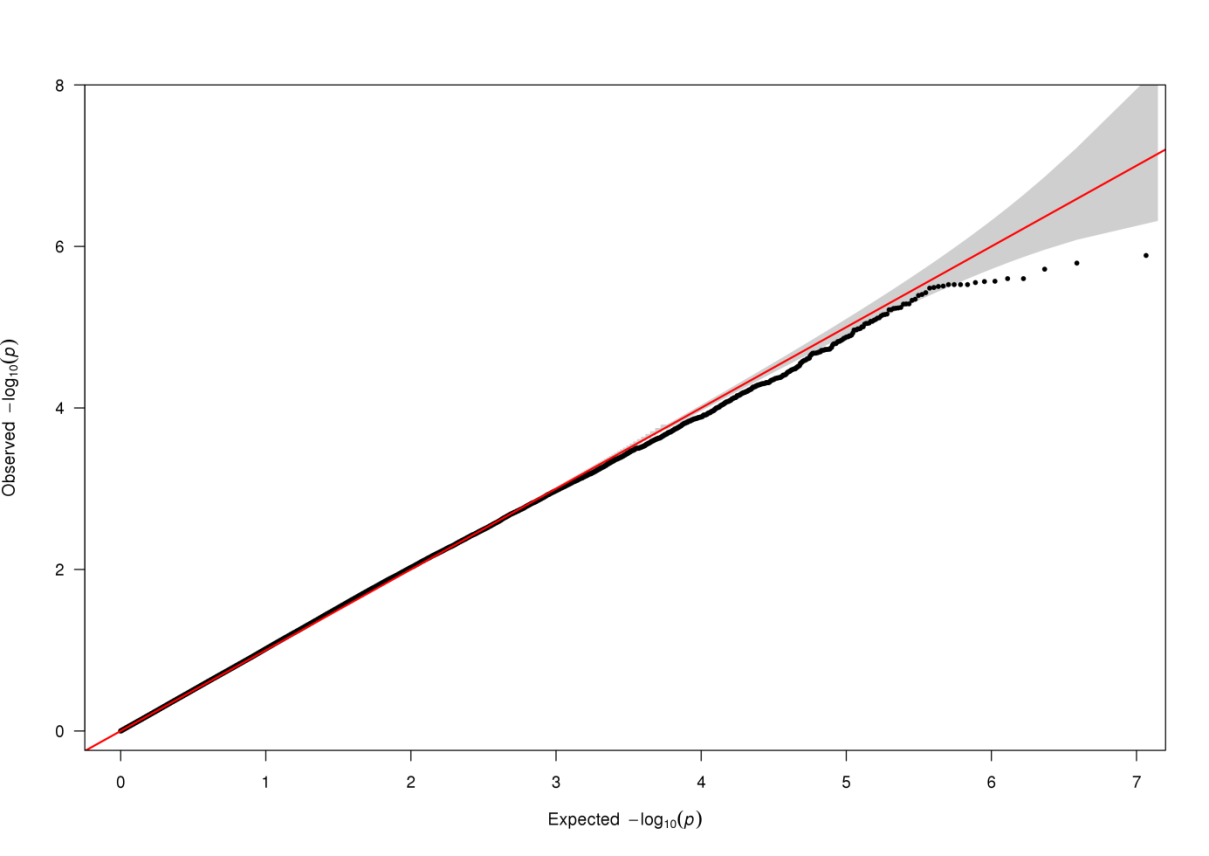


b.


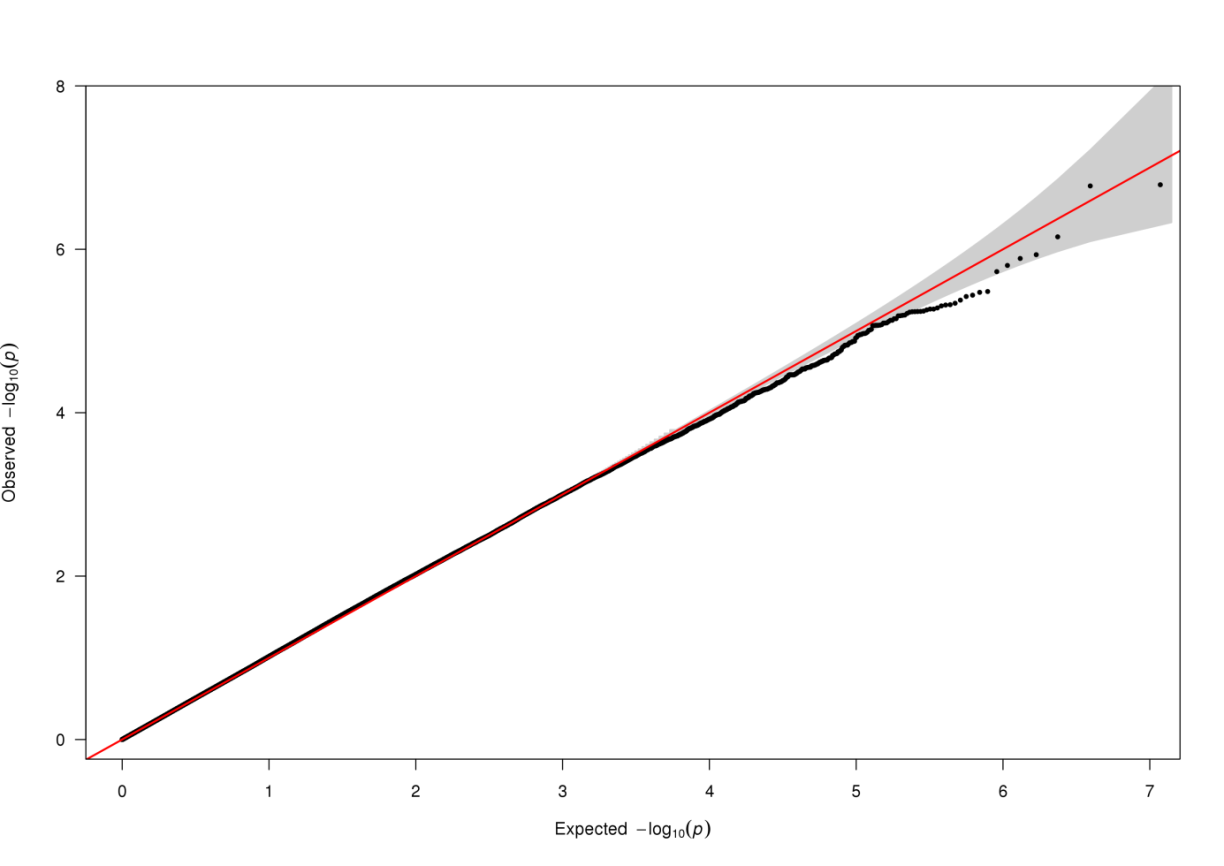


Figure S3: Regional plot for the top SNP in the analysis of initiation

**
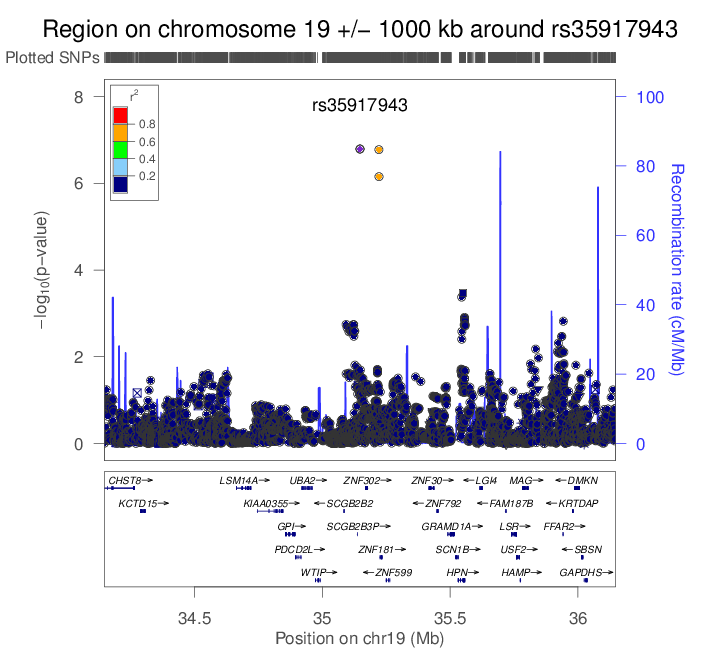
**

Figure S4: Lambda corrected Manhattan plots for the age of onset survival analysis. The analysis included same phenotyped sample from the Netherlands Twin Register (N=5148 individuals), with genotypes imputed based on (a) the 1000 Genomes project reference panel and based on (b) the Genome of the Netherlands (GoNL) project reference panel.

a.


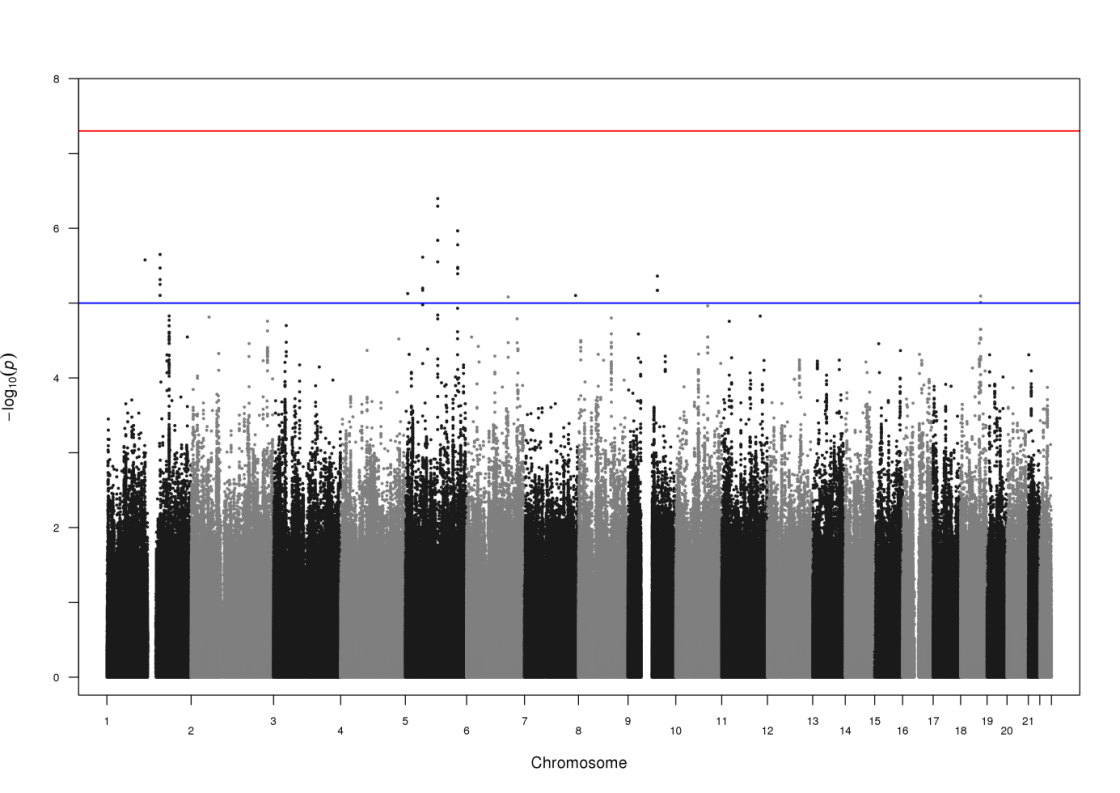


b.


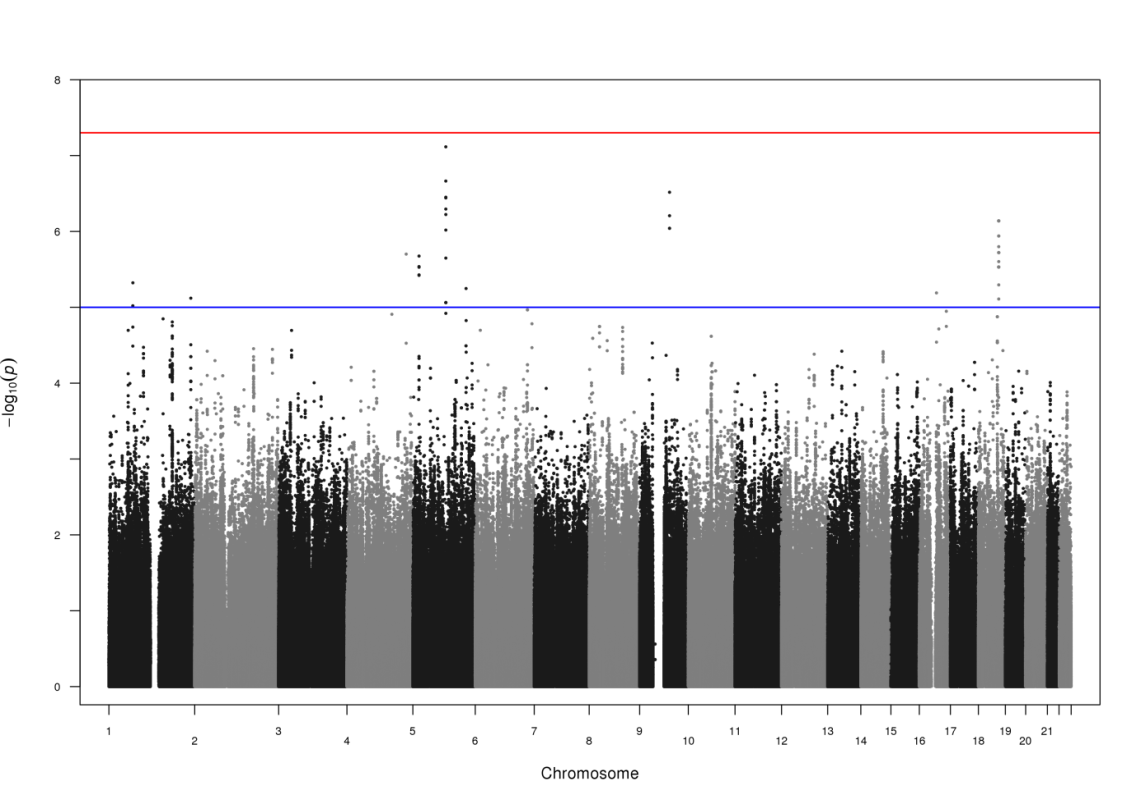


Figure S5: Lambda corrected quantile-quantile plots for the age of onset survival analysis. The analysis included same phenotyped sample from the Netherlands Twin Register (N=5148 individuals), with genotypes imputed based on (a) the 1000 Genomes project reference panel and based on (b) the Genome of the Netherlands (GoNL) project reference panel.

a.


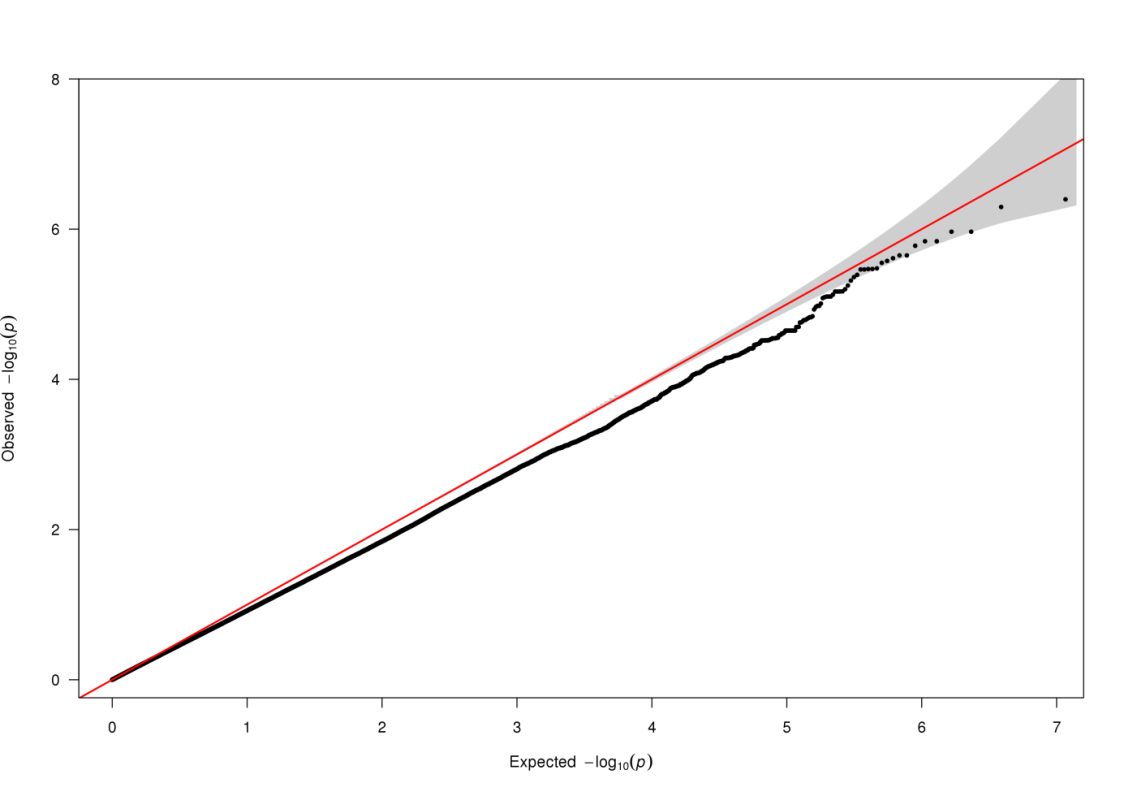


b.


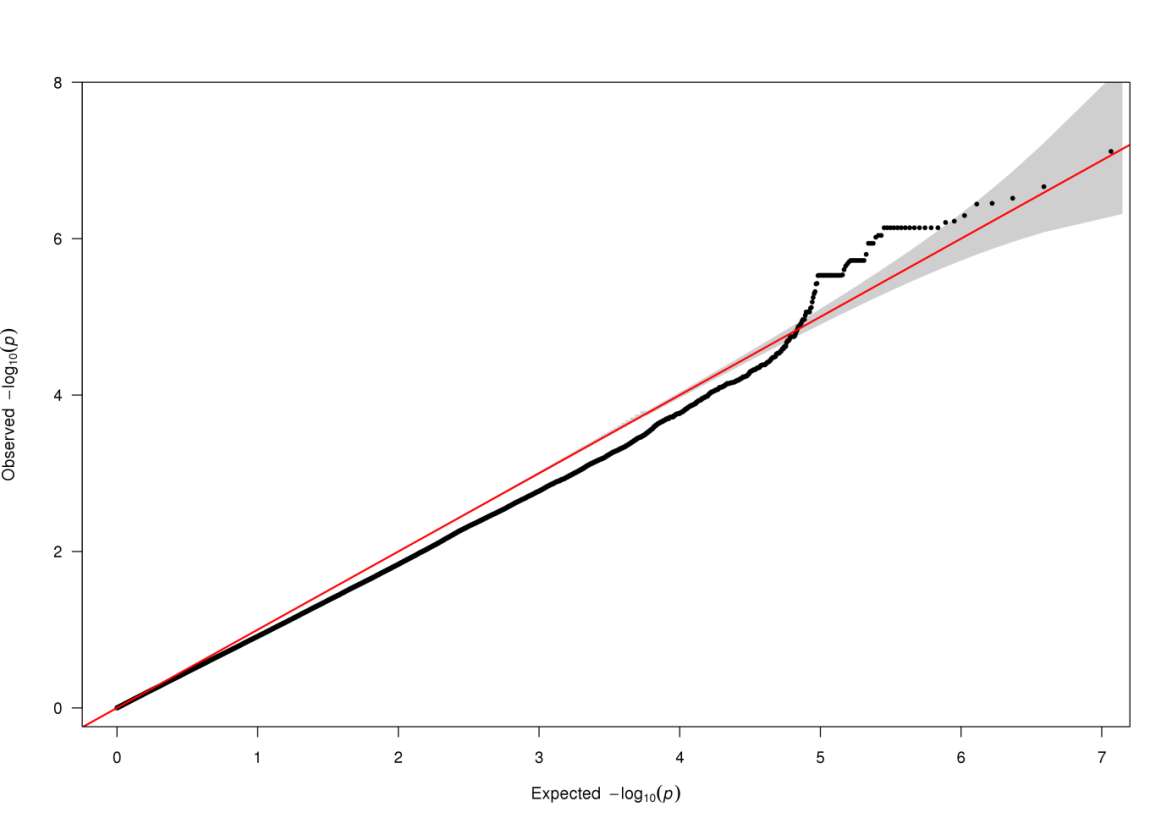


Figure S6: Regional plot around the top SNP in the survival analysis of age of onset


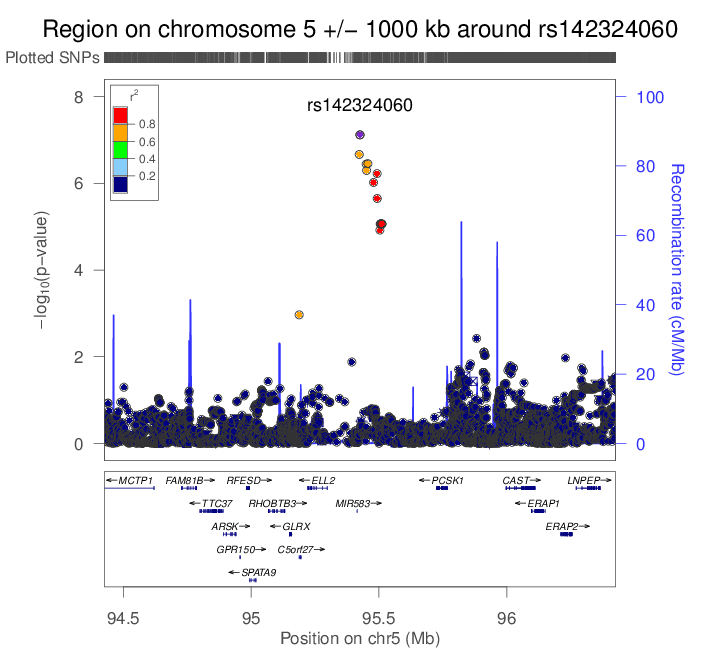

Supplement: Supplementary file 1 — Supplementary material 1 (DOCX 1311 kb) [file 10519_2015_9723_MOESM1_ESM.docx]
